# The role of BMI1 in assessing endometrial receptivity and its clinical implications

**DOI:** 10.3389/fmed.2026.1774247

**Published:** 2026-04-08

**Authors:** Meili Liu, Jiana Mao, Yanyan Yao, Lili Tu, Kemei Zhang

**Affiliations:** 1 Department of Obstetrics and Gynecology, The People’s Hospital of Fenghua Ningbo, Ningbo, China; 2 Reproductive Medicine Center, The First Affiliated Hospital of Ningbo University, Ningbo, China; 3 Department of Obstetrics and Gynecology, Yangming Hospital Affiliated to Ningbo University (Yuyao City People’s Hospital), Ningbo, China

**Keywords:** abortion, biomarker, BMI1, endometrial receptivity, infertility

## Abstract

**Objectives:**

Endometrial receptivity lacks robust biomarkers. Given its role in stabilizing the progesterone receptor, the epigenetic regulator BMI1 is a key candidate, yet its clinical potential is undefined. This study aimed to characterize BMI1’s expression and functional role in receptivity and evaluate its utility as a biomarker for predicting reproductive outcomes.

**Methods:**

Endometrial tissues were obtained from patients with elective (EA, *n* = 78) and spontaneous abortion (SA, *n* = 39). BMI1 levels, measured by immunohistochemistry (IHC), quantitative real-time PCR (qPCR), and western blot (WB), demonstrated diagnostic value by Receiver Operating Characteristic (ROC) analysis and predicted superior reproductive outcomes in a two-year infertile patient cohort (*n* = 50).

**Results:**

BMI1 expression was significantly downregulated at both the mRNA and protein levels in the SA compared to the EA group (*p* < 0.05), with predominant localization in the endometrial epithelium. This was accompanied by significant downregulation of upstream regulators (PR, E6AP) and downstream effectors (GATA6, NANOG) in the SA group, indicating impaired BMI1 pathway activity. ROC analysis confirmed the diagnostic utility of BMI1 for assessing receptivity. Clinical follow-up revealed that BMI1-positive patients had significantly higher rates of bio-chemical pregnancy, clinical pregnancy, and live birth compared to BMI1-negative patients.

**Conclusion:**

Our findings establish BMI1 as a critical regulator of endometrial receptivity, through its role in modulating progesterone signaling. The strong correlation between BMI1 status and pregnancy outcomes highlights its potential as a novel prognostic biomarker for infertility, offering new insights for diagnostic and therapeutic strategies in reproductive medicine.

## 1 Introduction

Infertility represents a significant global health challenge, conventionally defined as the failure to achieve a clinical pregnancy after 12 months of regular, unprotected intercourse (1). It affects an estimated 8%–12% of reproductive-aged couples worldwide, with prevalence rates climbing to 30% in certain developing regions. The growing incidence among younger populations further accentuates its substantial societal and healthcare burden (2). Successful embryo implantation is fundamental to the treatment of both infertility and pregnancy maintenance. This highly coordinated process requires an orchestrated molecular dialogue between a competent embryo and a receptive maternal endometrium (3, 4).

The critical state of endometrial receptivity—often termed the “implantation window”—denotes the limited period during which the uterine lining is conducive to blastocyst attachment and invasion (5). This synchrony between the embryo and endometrium is a prerequisite for implantation and is considered the dominant factor governing its success (6). Dysregulation of this process is implicated in the majority of implantation failures and is responsible for approximately 75% of pregnancy losses (7–9). Consequently, impaired endometrial receptivity is a major cause of infertility and early pregnancy loss, yet no specific or effective clinical indicators currently exist for its evaluation.

The establishment of endometrial receptivity is highly complex, involving an intricate interplay of hormones, signaling pathways, and transcriptional networks (10). Emerging evidence positions the polycomb protein B-cell-specific Moloney murine leukemia virus insertion region 1 (BMI1) as a critical upstream regulator of this pathway. As a core component of the Polycomb Repressive Complex 1 (PRC1), BMI1 is renowned for its roles in stem cell maintenance, cell cycle regulation, and epigenetic silencing. In reproductive medicine, studies have demonstrated that aberrantly low BMI1 expression is associated with compromised progesterone responsiveness, a defect particularly evident in endometrial tissue from patients with spontaneous miscarriage or recurrent pregnancy loss (11, 12). Progesterone signaling, mediated through its receptor (PR), is central to orchestrating endometrial differentiation and decidualization (13, 14). Mechanistically, BMI1 stabilizes the progesterone receptor via the E6AP-associated ubiquitination pathway, thereby potentiating progesterone signaling, which is essential for establishing a receptive endometrium. These findings suggest that BMI1 expression may serve as a biomarker for endometrial receptivity, warranting further investigation.

Despite these advances, the specific role of BMI1 in human endometrial receptivity, particularly in the contexts of implantation failure and early pregnancy loss, remains poorly delineated. Furthermore, its potential utility as a clinical biomarker has not been systematically evaluated (15, 16). Based on the evidence presented, we propose the hypothesis that BMI1 modulates endometrial receptivity through the PR signaling pathway, thereby influencing embryo implantation and potentially contributing to infertility. This study therefore aims to investigate the expression and functional role of BMI1 in endometrial tissues from patients experiencing spontaneous abortion (SA) compared to those undergoing elective termination (EA). Using a multifaceted approach, we assessed BMI1 expression, analyzed its associated signaling pathways, performed ROC-curve analyses to determine its diagnostic potential, and correlated BMI1 status with reproductive outcomes through clinical follow-up. Our findings aim to provide novel insights into the molecular regulation of endometrial receptivity and to evaluate BMI1 as a potential biomarker for predicting implantation success in infertile patients.

## 2 Materials and methods

### 2.1 Patient selection criteria

Participants were recruited from the Reproductive Medicine Center at the First Affiliated Hospital of Ningbo University and Obstetrics and Gynecology Department of Fenghua District People’s Hospital between 1 January 2023 and 31 December 2024. The study included a 2-year follow-up period, utilizing a cumulative data collection methodology per patient across all scheduled visits.

The inclusion criteria for SA/EA groups: (1) Confirmed intrauterine pregnancy ≤ 12 weeks (clinical ultrasound). (2) Age 20–50 years. (3) Provided informed consent with capacity to provide study data. Exclusion criteria : (1) concurrent medical conditions known to impair embryo implantation—including endometriosis, uterine anomalies, intrauterine adhesions, endometrial polyps, submucosal fibroids, or malignancies; (2) significant medical or surgical comorbidities (3) biochemical pregnancy or recurrent miscarriage; (4) known chromosomal abnormalities in either partner; and (5) refusal to provide informed consent.

The inclusion criteria for the infertility group: (1) infertility for ≥12 months despite regular unprotected intercourse; (2) age 20–50 years; and (3) provision of informed consent with capacity to provide necessary information. Exclusion criteria included: (1) known absolute female infertility factors, such as uterine malformation, severe intrauterine adhesions, malignancies, or ovarian failure; (2) severe internal medical or surgical comorbidities; (3) known severe male-factor infertility; and (4) refusal to sign the informed consent form.

### 2.2 Tissue preparation

Endometrial tissue specimens were collected under sterile conditions during surgical procedures and promptly transferred to the laboratory. Following collection, tissues were rinsed three times in phosphate-buffered saline (PBS) and subsequently divided into three portions for distinct downstream analyses. One portion was processed immediately for protein and RNA extraction to perform enzyme-linked immunosorbent assay (ELISA), Western blot (WB), and quantitative real-time polymerase chain reaction (qRT-PCR). A second portion was fixed, paraffin-embedded, and sectioned at 4–5 μm thickness for immunohistochemical (IHC) evaluation. The remaining tissue was snap-frozen and stored at −80 °C for potential future use.

### 2.3 Immunohistochemistry

Tissue sections were immunostained overnight at 4 °C using anti-BMI1 primary antibody (1:200 dilution, Cell Signaling, United States). Antigen retrieval was performed using citrate buffer treatment, followed by incubation one hour at 37 °C with a horseradish peroxidase (HRP)-conjugated polymeric anti-rabbit secondary antibody. Sections were then counterstained with hematoxylin. Each experiment included both negative and positive controls. For analysis, five randomly selected fields per sample were evaluated at magnification × 400 under an optical microscope (Leica DM500 ICC50, Leica).

### 2.4 RNA extraction and real-time polymerase chain reaction

Total RNA was isolated from tissue samples using TRIzol Reagent (Invitrogen, United States) following the manufacturer’s protocol. RNA concentration was measured with a Thermo spectrophotometer. For cDNA synthesis, 1 μg of total RNA was reverse transcribed using the RevertAid First Strand cDNA Synthesis Kit (Thermo Scientific, United States). Quantitative RT-PCR was carried out in 25-μL reactions containing: 12.5 μL of 2× SYBR Green PCR Master Mix (TaKaRa, Japan), 0.5 μL each of forward and reverse primers (10 μM), 1 μL cDNA template, and 10.5 μL nuclease-free water. GAPDH served as the housekeeping gene for normalization. The thermal cycling protocol consisted of: initial denaturation at 95 °C for 10 min; 40 cycles of 95 °C for 15 s, 60 °C for 15 s, and 72 °C for 30 s; followed by a dissociation curve analysis (95 °C for 15 s, 60 °C for 30 s, and 95 °C for 15 s). All samples included three biological replicates, with each PCR reaction performed in triplicate. Data analysis was conducted using Eppendorf RealPlex 2 software. Primer sequences for target genes are provided in Supplementary Table 1.

### 2.5 Western blotting

Total protein was extracted from tissue samples using RIPA lysis buffer supplemented with 1% PMSF. Protein concentrations were measured using a BCA protein assay kit (Beyotime, China). For immunoblotting, proteins were resolved by 15% SDS-PAGE and subsequently transferred to PVDF membranes (Merck Millipore, GER). The membranes were blocked with 5% non-fat milk in TBST for 1 h at room temperature, followed by overnight incubation at 4 °C with primary antibodies, including anti-BMI1 (1:10000, abcam, United States). After washing, membranes were incubated for 1 h at room temperature with HRP-conjugated secondary antibodies (1:1000, Beyotime, China). Protein bands were visualized using the BeyoECL Plus Kit (Beyotime), and band intensities were quantified using Quantity One software (version 4.62, Bio-Rad).

### 2.6 ELISA

First, coating the plate with diluted capture antibodies, followed by an incubation period to allow adsorption. Next, after discarding the liquid, the plate is washed three times with PBS to remove any unbound antibodies. To prevent non-specific binding, a blocking solution is added and incubated at 37 °C for 1 h. Following another wash step, gradient-diluted endometrial tissue lysates samples are introduced and incubated at 37 °C for a further hour, enabling the target protein to bind to the immobilized capture antibodies. The plate is washed again three times before adding an HRP-conjugated detection antibody, which is incubated for another hour at 37 °C. After five thorough washes to ensure the removal of any unbound detection antibody, a substrate solution is added, and the plate is incubated in the dark at room temperature for 30 min to allow for color development. The reaction is then stopped with a stop solution, and the absorbance is immediately measured at 450 nm using a microplate reader. Finally, the target protein concentration in each sample is calculated by interpolating the absorbance values from a standard curve generated using reference standards.

### 2.7 Statistical analysis

All statistical analyses were conducted using SPSS and GraphPad Prism. Normality and homogeneity of variance were assessed for each dataset. Normally distributed data were analyzed using Student’s *t*-test, while non-parametric data were evaluated with the Mann-Whitney U test (independent samples rank sum test). Statistical significance was defined as *p* < 0.05 for all comparisons.

## 3 Results

### 3.1 Clinical characteristics of the study population

The study included a total of 117 participants, comprising 78 in the EA group and 39 women in the SA group. Demographic analysis revealed comparable characteristics between the two groups, with no statistically significant differences in key parameters. The mean age was similar between groups (31.32 ± 6.199 years for EA vs. 31.54 ± 5.844 years for SA, *p* > 0.05), as was body mass index (21.54 ± 2.34 vs. 22.50 ± 3.42, respectively). Reproductive history showed comparable median numbers of previous pregnancies (3 vs. 2) and deliveries between groups. Educational attainment, ranging from elementary to graduate level, was similarly distributed across both cohorts. These findings confirm balanced baseline characteristics between study groups, supporting meaningful comparison of subsequent experimental outcomes (Table 1).

**TABLE 1:** Baseline demographic and clinical characteristics of study participants.

Characteristics	Elective abortion (*n* = 78)	Spontaneous abortion (*n* = 39)	*P*-value
Age (mean ± SD)	31.32 ± 6.199	31.54 ± 5.844	0.956
BMI (kg/m^2^) (mean ± SD)	21.54 ± 2.34	22.50 ± 3.42	0.198
Previous number of pregnancies (median)	3 (0–6)	2 (0–7)	0.853
Educational attainment *n* (%)	–	–	0.122
Graduate student	1.3	0	–
Undergraduate degree	12.8	10.1	–
Junior college	20.5	23.1	–
High school or vocational school	34.6	12.9	–
Middle school	23.1	41	–
Primary school	7.7	12.9	–

Comparison of age, body mass index (BMI), reproductive history, and educational background between elective abortion (EA) and spontaneous abortion (SA) groups. No statistically significant differences were observed, confirming balanced baseline characteristics.

### 3.2 Expression of BMI1 in the endometrium of patients with abortion

**FIGURE 1:**
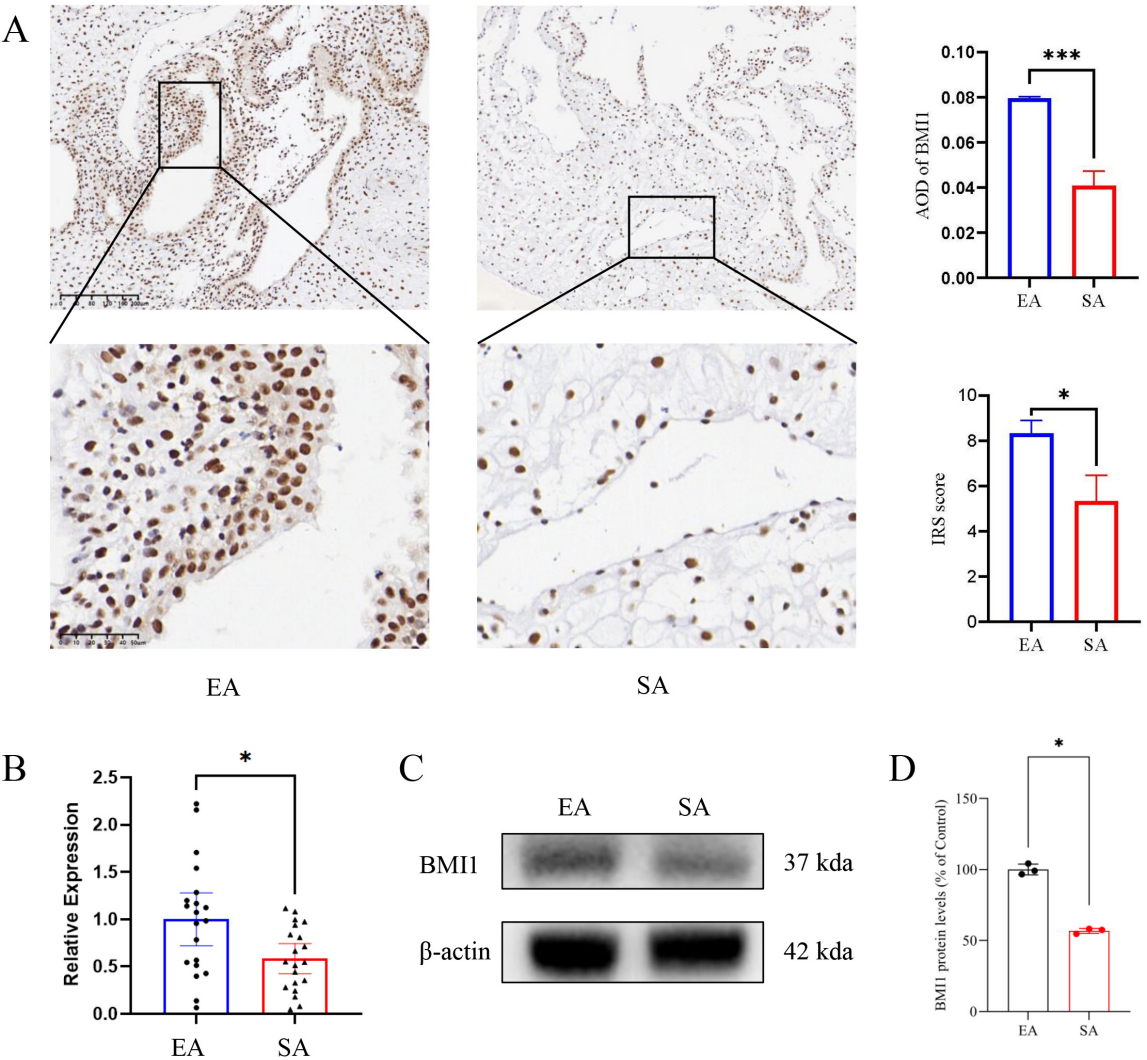
BMI1 expression is significantly downregulated in endometrial tissues from spontaneous abortion (SA) group. **(A)** Representative immunohistochemical staining (IHC) and semi-quantitative analysis show strong BMI1 expression localized predominantly in the epithelial layer of the endometrium in elective termination (EA) group, with markedly reduced expression in SA group (*n* = 3). **(B)** Quantitative real-time PCR (qPCR) analysis confirms a significant reduction in BMI1 mRNA levels in SA compared to EA (*n* = 20). **(C,D)** Western blot (WB) shows significantly decreased BMI1 protein expression in SA tissues (*n* = 3). (*p < 0.05, ***p < 0.001).

To investigate BMI1 expression patterns, endometrial tissues from both study groups underwent IHC staining, qPCR, and WB analysis. IHC revealed predominant BMI1 localization in the endometrial epithelial layer, with visibly reduced expression in the spontaneous abortion group compared to controls (Figure 1A). BMI1 expression was also detected in a subset of mesenchymal cells. Comparative analysis revealed no statistically significant difference in BMI1 levels between the two groups (Supplementary Figure 1). Subsequent qPCR analysis demonstrated significant downregulation of BMI1 mRNA levels in SA cases (*p* < 0.05, Figure 1B), a finding corroborated by WB showing corresponding decreases in BMI1 protein expression (p < 0.05) (Figures 1C, D).

### 3.3 Suppression of the BMI1 pathway in SA

**FIGURE 2:**
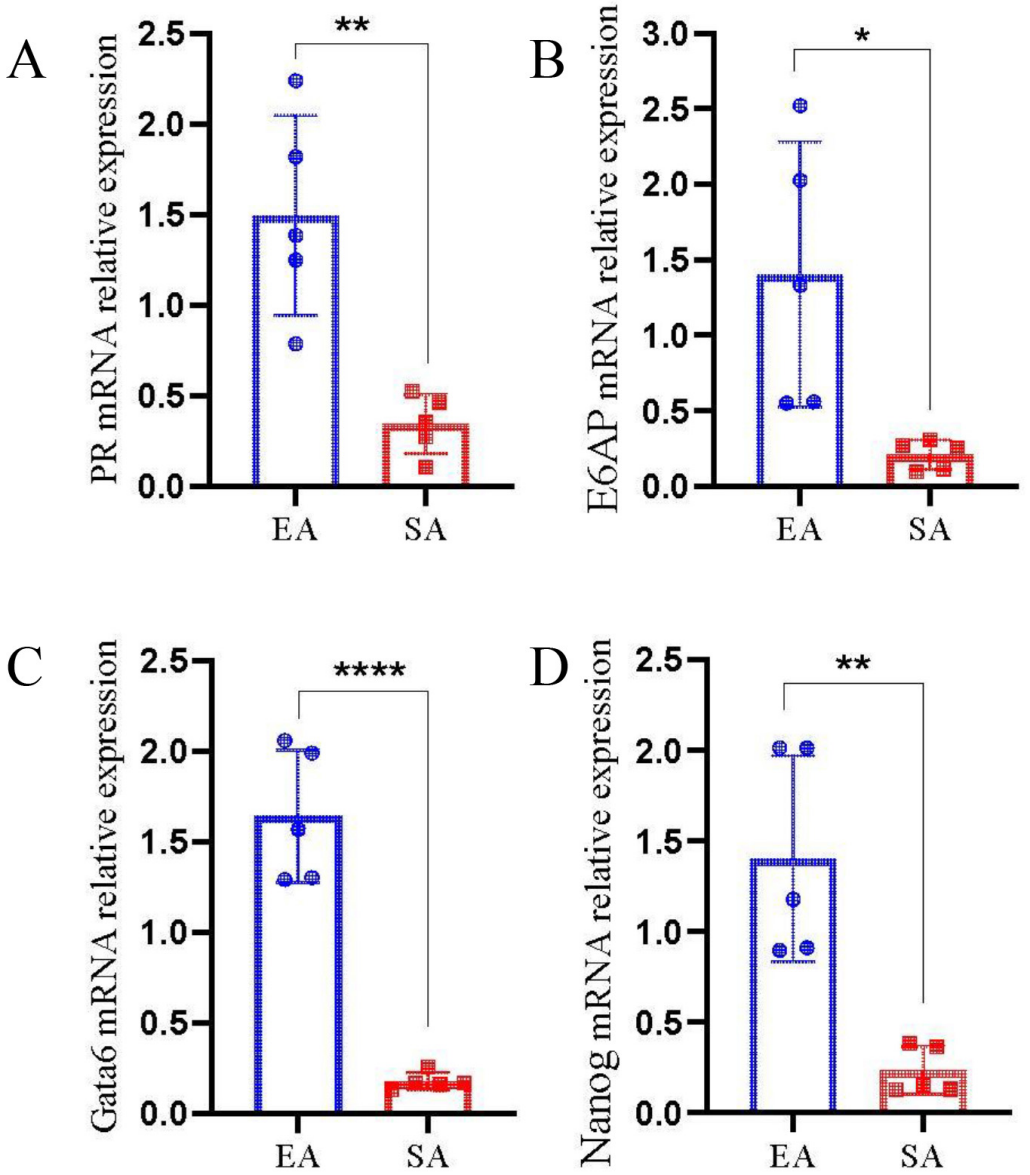
BMI1 signaling pathway is suppressed in SA. Quantitative real-time polymerase chain reaction (qRT-PCR) analysis reveals significantly reduced mRNA expression of upstream regulators PR **(A)** and E6AP **(B)**, as well as downstream targets GATA6 **(C)** and NANOG **(D)**, in spontaneous abortion (SA) compared to elective termination (EA) tissues. (*p < 0.05, **p < 0.01, ****p < 0.0001).

To further assess BMI1 signaling pathway activity, the mRNA expression levels of pathway-associated molecules were analyzed via qRT-PCR. The results demonstrated significantly downregulated mRNA expression of upstream regulators PR (Figure 2A) and E6AP (Figure 2B) in SA compared to EA tissues. Consistent with these upstream alterations, downstream effectors GATA6 (Figure 2C) and NANOG (Figure 2D) also exhibited statistically significant downregulation in SA (*p < 0.05). These findings collectively suggest suppression of the BMI1 signaling pathway in SA.

### 3.4 BMI1 as a biomarker for endometrial receptivity assessment

**FIGURE 3:**
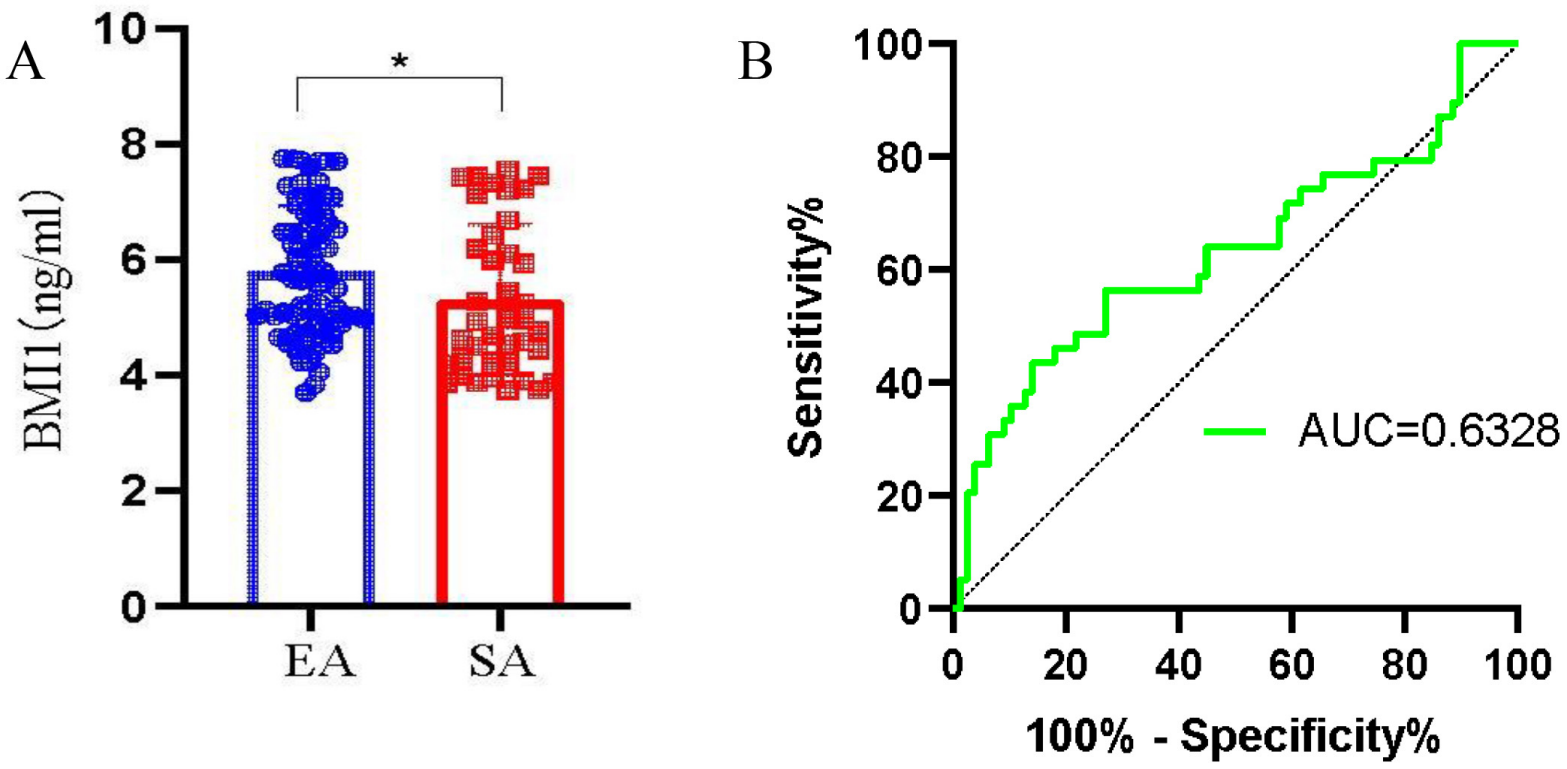
BMI1 shows diagnostic potential as a biomarker for endometrial receptivity. **(A)** Enzyme-linked immunosorbent assay (ELISA) quantification of BMI1 protein levels in endometrial tissue shows significantly lower expression in spontaneous abortion (SA) (*n* = 39) compared to elective termination (EA) (*n* = 78). **(B)** The area under the curve is 0.6328, indicating that the diagnostic performance distinguishes between receptive (EA) and non-receptive (SA) states, albeit with limited discriminative efficacy. (*p < 0.05).

Subsequently, to evaluate the potential of BMI1 as a biomarker for endometrial receptivity, we performed ELISA and clinical follow-up studies. Initial ELISA quantification revealed differential BMI1 expression in endometrial tissues from SA and EA cases (Figure 3A). Then, ROC curve analysis established the diagnostic cutoff value for BMI1 in assessing endometrial receptivity (Figure 3B). The AUC for BMI1 was 0.6328, suggesting a potential association with endometrial receptivity. Based on this threshold, subsequent clinical analyses were conducted to evaluate the utility of BMI1 as a biomarker for receptivity assessment. In our cohort of 50 infertility patients, stratification by BMI1 expression levels (cutoff-based) yielded BMI1-positive (*n* = 32) and BMI1-negative (*n* = 18) groups. Comparative analysis showed no significant age difference (30.59 ± 3.93 vs. 32.28 ± 4.38 years) or educational background between groups, but revealed significantly lower BMI in BMI1-positive patients (21.63 ± 2.68 vs. 23.84 ± 3.17 kg/m^2^, *p* < 0.05). Endometrial characteristics, including polyp presence, menstrual cycle phase, and CD38/CD138 expression patterns, showed no intergroup differences (Table 2).

**TABLE 2:** Clinical characteristics of infertility patients stratified by BMI1 expression.

Characteristic	BMI1-positive expression group (*n* = 32)	BMI1-negtive expression group (*n* = 18)	*P*-value
Age (mean ± SD)	30.59 ± 3.93	32.28 ± 4.38	0.149
BMI	21.63 ± 2.68	23.84 ± 3.17	0.012*
Educational attainment (*n*,%)	–	–	0.062
Bachelor’s degree	13 (40.6%)	4 (22.2%)	–
Associate degree/college diploma	12 (37.5%)	5 (27.8%)	–
Senior high school or secondary vocational school	2 (6.3%)	3 (16.7%)	–
Junior high school	5 (15.6%)	6 (33.3%)	–
Presence of endometrial polyp	12 (37.5%)	6 (33.3%)	0.768
Absence of endometrial polyp	20 (62.5%)	12 (66.7%)	
Proliferative phase (menstrual cycle)	27 (84.4%)	14 (77.8%)	0.842
Secretory phase (menstrual cycle)	5 (15.6%)	4 (22.2%)	
CD38/CD138 double-negative	8 (25.0%)	4 (22.2%)	1
CD38/CD138 positive	24 (75.0%)	14 (77.8%)	

Comparison of age, BMI, endometrial features, and immunohistochemical markers (CD38/CD138) between BMI1-positive and BMI1-negative infertility patients. The only significant difference was observed in BMI, with BMI1-positive patients having a lower average BMI. * indicates that the difference is statistically significant.

**FIGURE 4:**
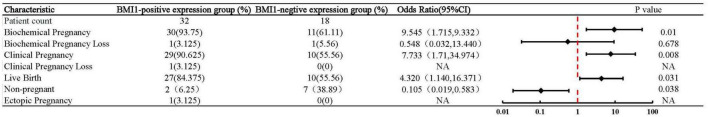
Reproductive outcomes stratified by BMI1 expression status. Following a 2-year clinical follow-up of 50 infertility patients, those classified as BMI1-positive exhibited significantly higher rates of biochemical pregnancy, clinical pregnancy, and live birth compared to BMI1-negative individuals. Conversely, BMI1-negative patients had a higher rate of persistent infertility. No significant difference was observed in the rate of biochemical pregnancy loss between the two groups.

Furthermore, after a 2-year clinical follow-up of 50 infertility patients, significant differences in reproductive outcomes were observed between BMI1-positive and BMI1-negative group. The BMI1-positive group demonstrated higher rates of biochemical pregnancy (93.75% vs. 61.11%), clinical pregnancy (90.625% vs. 55.56%), and live birth (84.375% vs. 55.56%) compared to the BMI1-negative group (Supplementary Table 2). Conversely, the BMI1-negative group showed a higher rate of persistent infertility (38.89% vs. 6.25%). No significant difference was found between the groups in rates of biochemical pregnancy loss (Figure 4).

Given that obesity is an established independent risk factor for infertility, and a significant difference in body mass index (BMI) was observed between the BMI1-positive and BMI1-negative groups, we performed multivariate logistic regression to assess whether BMI1 retains predictive value independent of body weight. The analysis confirmed that BMI1 expression status predicts pregnancy outcomes independently of BMI. These findings indicate that BMI1 may serve as an independent biomarker of endometrial receptivity, and its clinical relevance is not confounded by the weight differences present in this cohort (Table 3).

**TABLE 3:** Logistic regression analysis of the associations between BMI1 expression, BMI, and pregnancy outcomes.

Outcome variable	Predictor variable	Odds ratio (OR)	95% confidence interval (CI)	*P*-value	Model AUC (SE)	Model pseudo-R^2^ (Tjur)
Biochemical pregnancy	BMI1	6.046	1.021–50.40	<0.05*	0.7007 (0.1156)	0.09787
	BMI	0.9847	0.7423–1.318	>0.05	–	–
Clinical pregnancy	BMI1	3.414	0.8184–15.27	>0.05	0.6700 (0.0930)	0.08183
	BMI	0.9527	0.7537–1.207	>0.05	–	–
Live birth	BMI1	0.2735	0.06663–0.9999	<0.05*	0.6698 (0.0779)	0.07725
	BMI	0.9368	0.7487–1.154	>0.05	–	–
Persistent infertility	BMI1	7.548	1.418–59.07	<0.05*	0.7683 (0.0983)	0.1821
	BMI	1.128	0.8630–1.491	>0.05	–	–

In the biochemical pregnancy model, the odds ratio (OR) for BMI1 was 6.046 (95% CI: 1.021–50.40, *P* < 0.05). In the clinical pregnancy model, the OR for BMI1 was 3.414 (95% CI: 0.8184–15.27, *P* = 0.091). For the live birth model, BMI1 showed an OR of 0.2735 (95% CI: 0.06663–0.9999, *P* < 0.05). In the persistent infertility model, the OR for BMI1 increased further to 7.548 (95% CI: 1.418–59.07, *P* < 0.05). By contrast, BMI did not demonstrate a statistically significant effect in any of these models (all *P* > 0.05. * indicates that the difference is statistically significant).

## 4 Discussion

This study systematically explores the biological function of BMI1 in modulating endometrial receptivity and, through an integrative analysis of molecular mechanisms and clinical data, assesses its potential as a predictive biomarker for pregnancy outcomes in infertile women. Our results not only identify its differential expression patterns in SA and EA but also reveal significantly improved reproductive performance in patients with BMI1-positive endometrial status. These findings offer valuable theoretical insights and potential therapeutic targets for clinical applications in reproductive medicine.

The observed downregulation of BMI1 in SA endometrial tissue is consistent with its well-characterized role. Functioning as an essential subunit of PRC1, BMI1 maintains PR stability through E6AP, thereby ensuring proper progesterone signaling - a fundamental requirement for endometrial receptivity (14, 15, 17). In our cohort, the baseline characteristics were closely matched between the SA and EA groups indicating that differences in BMI1 expression likely contribute to disparate pregnancy outcomes. BMI1 was significantly downregulated in SA endometrial tissues at both transcriptional and translational levels, with expression localized predominantly to the endometrial epithelium. Mechanistically, BMI1 stabilizes the PR signaling axis through E6AP, facilitating the proliferation-to-differentiation switch (PDS) crucial for endometrial receptivity (17–19). Supporting this, our qRT-PCR data demonstrated parallel downregulation of up-stream regulators (PR and E6AP) and downstream effectors (GATA6 and NANOG) in the SA group, suggesting systemic impairment of this pathway. Our results further demonstrate the extensive regulatory impact of BMI1 on downstream transcriptional effectors, particularly the pluripotency factors GATA6 and NANOG, which play indispensable roles in endometrial differentiation and successful embryo implantation (20–22). GATA6 is a critical marker of the primitive endoderm during embryogenesis, where it directs endodermal lineage specification. In the endometrium, GATA6 is predominantly expressed in stromal cells. Its elevated expression has been shown to attenuate hormonal sensitivity and disrupt the expression of key genes necessary for normal decidualization of endometrial stromal cells, thereby contributing to impaired uterine receptivity and representing one molecular mechanism underlying “progesterone resistance” (23). In contrast, a recent single-cell sequencing study identified a population of peritoneal-derived macrophages expressing GATA6 (GATA6^+^ LPMs) that migrate to sites of endometrial injury. These cells secrete IL-33, which suppresses the differentiation of stromal cells into myofibroblasts and exhibits anti-fibrotic activity (21). This finding indicates that under physiological conditions, GATA6 expression may be associated with the function of non-epithelial immune cells rather than serving as a direct marker of epithelial cells. NANOG functions as a core transcriptional regulator responsible for maintaining pluripotency and self-renewal while inhibiting premature lineage commitment. Although NANOG is intrinsically linked to pluripotent states, the gene networks it governs are essential for establishing normal endometrial receptivity. Within the endometrium, NANOG is primarily localized to epithelial cells, and its expression correlates with cellular proliferation, tissue repair, and certain pathological conditions. Acting as a master regulator, NANOG plays a pivotal role in coordinating endometrial cycle progression and modulating functional gene networks. It operates within a complex transcriptional circuitry, alongside factors such as GATA6, to regulate a broad spectrum of genes critical for endometrial receptivity (24). This study provides the systematic delineation of the coordinated regulatory network comprising BMI1, PR, and E6AP in maintaining endometrial homeostasis. Our findings demonstrate that disruption of this axis, as evidenced by the significant downregulation of BMI1 and its associated signaling components in the SA cohort, leads to impaired decidualization and consequent receptivity failure. These mechanistic observations are substantiated by experimental evidence and align precisely with established literature emphasizing the central role of PR signaling in pregnancy establishment and maintenance (25, 26). Our findings provide a novel theoretical framework for under-standing the molecular mechanisms underlying embryo implantation failure, offering potential diagnostic and therapeutic implications for infertility management.

This study provides molecular validation of the “progesterone-resistance” hypothesis (25, 27). We establish the clinical evidence linking aberrant BMI1 expression to compromised endometrial receptivity, with BMI1-positive patients exhibiting significantly higher biochemical pregnancy rates and live birth rates. Mechanistically, the observed effects may be attributed to multifunctional regulation mediated through some pathways, cell cycle modulation via activating CDK4/6-p53 signaling (28, 29); tissue remodeling through EMT-driven E-cadherin suppression and PI3K/AKT-mediated Slug/N-cadherin upregulation (30–32); and stress potentiation via p16/p19 pathways contributing to infertility. While the role of BMI1 in stem cell integration is conserved (33), species-specific differences in miR-194/200c binding sites within the human BMI1 3’-UTR necessitate caution, which is absent in murine homologs (31, 34). Our breakthrough establishes BMI1’s dual utility as a novel infertility biomarker and therapeutic target. While compounds like PTC-209 and PTC-028 demonstrate BMI1-inhibitory efficacy in oncology contexts (31, 34), their precise effects on uterine receptivity and clinical applicability in infertility management warrant rigorous investigation.

While this study provides compelling evidence for the role of BMI1 in endometrial receptivity, several limitations merit consideration. First, the current sample size yielded robust initial findings. The result of AUC is significantly greater than random chance, but falls below the conventional threshold for strong discriminative power. This indicates a weak-to-moderate association with endometrial receptivity, suggesting limited utility as a standalone diagnostic marker. Second, the mechanistic relationship between BMI1 and PR signaling, particularly the isoform-specific interactions of PR, remains incompletely characterized. Third, longitudinal profiling of BMI1 expression dynamics across the menstrual cycle is essential to optimize its utility as a cycle-dependent biomarker. Moreover, during cohort selection, cases with known fetal chromosomal aneuploidy were excluded. However, in routine clinical practice, such cytogenetic testing is not universally performed, and suitable chorionic villus or fetal tissue is not always obtained following spontaneous abortion. Therefore, the 117 tissue samples included in this analysis comprise cases both with and without available karyotype results. Our team’s ongoing research continues to evaluate the potential of integrating BMI1 into other factors and its association with specific gestational states, which may enhance its clinical applicability. Future work will include larger multi-center cohorts are required to establish definitive diagnostic thresholds for BMI1 expression. Future investigations should prioritize therapeutic strategies targeting BMI1, including pharmacological activators of BMI1 signaling. Preclinical models employing conditional BMI1 knockout or overexpression systems would further elucidate its spatiotemporal regulation of endometrial biology.

## 5 Conclusion

This work establishes BMI1 as both a critical regulator of endometrial receptivity and a promising biomarker for predicting pregnancy outcomes in infertility. By integrating molecular mechanisms, clinical correlations, and evolutionary-conserved functions, we demonstrate the centrality of BMI1 in reproductive success and its translational potential. These insights provide a foundation for novel diagnostic frameworks and targeted therapies in reproductive medicine, ultimately advancing clinical management of infertility and recurrent pregnancy loss.

## Data Availability

The raw data supporting the conclusions of this article will be made available by the authors, without undue reservation.

## Supplementary material

Supplementary Table 1 Primer sequences for target genes.

Supplementary Table 2 Live birth outcomes stratified by conception methods in BMI1-positive and BMI1-negative groups.

Supplementary Figure 1 The expression of BMI1 in mesenchymal cells.
