# Supplementary figures and images for: The role of BMI1 in assessing endometrial receptivity and its clinical implications
Source: Front Med (Lausanne). 2026 Apr 8;13:1774247. doi: 10.3389/fmed.2026.1774247 (PMC13099878; doi:10.3389/fmed.2026.1774247)

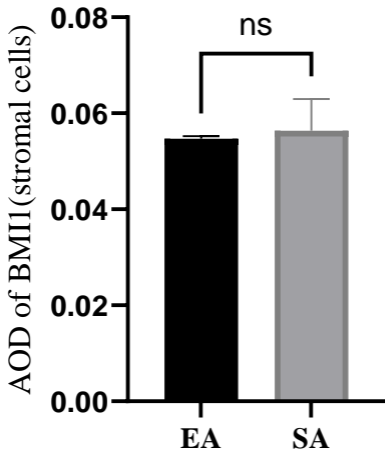

Supplement: Supplementary Figure 1 — The expression of BMI1 in mesenchymal cells. [file Data_Sheet_1.pdf]
